# Deficiency of phyto-available sulphur, zinc, boron, iron, copper and manganese in soils of India

**DOI:** 10.1038/s41598-021-99040-2

**Published:** 2021-10-05

**Authors:** Arvind Kumar Shukla, Sanjib Kumar Behera, Chandra Prakash, Ajay Tripathi, Ashok Kumar Patra, Brahma Swaroop Dwivedi, Vivek Trivedi, Ch. Srinivasa Rao, Suresh Kumar Chaudhari, Soumitra Das, Anil Kumar Singh

**Affiliations:** 1 ICAR-Indian Institute of Soil Science, Bhopal, Madhya Pradesh 462038 India; 2 ICAR-National Bureau of Soil Survey and Land Use Planning, Nagpur, Maharashtra 440033 India; 3 ICAR-Indian Agricultural Research Institute, New Delhi, 110012 India; 4 ICAR-National Academy of Agricultural Research Management, Hyderabad, Telangana 500030 India; 5 Indian Council of Agricultural Research, New Delhi, 110012 India; 6 International Zinc Association, New Delhi, 110062 India

**Keywords:** Biogeochemistry, Environmental sciences, Solid Earth sciences

## Abstract

Nutrient deficiencies in soil–crop contexts and inappropriate managements are the important reasons for low crop productivity, reduced nutritional quality of agricultural produce and animal/human malnutrition, across the world. The present investigation was carried out to evaluate nutrient deficiencies of sulphur (S) and micronutrients [zinc (Zn), boron (B), iron (Fe), copper (Cu) and manganese (Mn)] in agricultural soils of India for devising effective management strategies to achieve sustainable crop production, improved nutritional quality in crops and better animal/human health. A total of 2,42,827 surface (0–15 cm depth) soil samples were collected from agriculture fields of 615 districts lying in 28 states of India and were analysed for available S and micronutrients concentration. The study was carried out under the aegis of All India Coordinated Research Project on Micro- and Secondary-Nutrients and Pollutant Elements in Soils and Plants. The mean concentrations were 27.0 ± 29.9 mg kg^−1^ for available S, 1.40 ± 1.60 mg kg^−1^ for available Zn and 1.40 ± 4.70 mg kg^−1^ for available B, 31.0 ± 52.2 mg kg^−1^ for available Fe, 2.30 ± 3.50 mg kg^−1^ for available Cu and 17.5 ± 21.4 mg kg^−1^ for available Mn. There were variable and widespread deficiencies of S and micronutrients in different states. The deficiencies (acute deficient + deficient + latent deficiency) of S (58.6% of soils), Zn (51.2% of soils) and B (44.7% of soils) were higher compared to the deficiencies of Fe (19.2% of soils), Cu (11.4% of soils) and Mn (17.4% of soils). Out of 615 districts, > 50% of soils in 101, 131 and 86 districts were deficient in available S, available Zn and available B, respectively. Whereas, > 25% of soils in 83, 5 and 41 districts had deficiencies of available Fe, available Cu and available Mn, respectively. There were occurrences of 2-nutrients deficiencies such S + Zn (9.30% of soils), Zn + B (8.70% of soils), S + B (7.00% of soils) and Zn + Fe (5.80% of soils) to a greater extent compared to the deficiencies of Zn + Mn (3.40% of soils), S + Fe (3.30% of soils), Zn + Cu (2.80% of soils) and Fe + B (2.70% of soils). Relatively lower % of soils were deficient in 3-nutrients (namely S + Zn + B, S + Zn + B and Zn + Fe + B), 4-nutrients (namely Zn + Fe + Cu + Mn) and 5-nutrients (namely Zn + Fe + Cu + Mn + B) simultaneously. The information regarding the distribution of deficiencies of S and micronutrients (both single and multi-nutrients) could be used by various stakeholders for production, supply and application of right kind of fertilizers in different districts, states and agro-ecological regions of India for better crop production, crop nutritional quality, nutrient use efficiency, soil health and for tackling human and animal malnutrition.

## Introduction

Soil is linked to wellness of human being and animals via agriculture and produces obtained from agriculture^1^. Crop plants take desired nutrients, primarily from soil, for their growth and development^2–4^. The deficiency of phyto-available (hereafter referred as available) nutrients in soils adversely affect soil health, crop production, nutritional quality of agricultural produces and in turn human/animal health^5,6^. Along with deficiencies of macronutrients namely, nitrogen (N), phosphorus (P) and potassium (K) deficiency, several researchers of the world reported the deficiency of micronutrients (zinc (Zn), boron (B), iron (Fe), copper (Cu) and manganese (Mn)) and sulphur (S) in various soils under cultivation^7–11^. The appearance of deficiencies of micronutrients in different soils is mainly due to enhanced cropping intensity, high-yielding cultivars of various crops, increase used NPK fertilisers with nil or less micronutrients contents, nil or very less application of organic manures and better soil and plant analysis technologies^12^. The proportion of deficiencies of S and micronutrients in different arears such as different districts and states of a country is different due to differences in soils, climates, crops and crop management options^5,13^. Soils with micronutrients deficiencies produce crops with low concentration of micronutrients^11,14^. Consumption of foods obtained from such crops lead to poor health of animal and human, because of micronutrients malnutrition. However, effective management of micronutrients, namely soil and foliar application of micronutrients, in various soil–crop situations could help in alleviating micronutrients deficiency in soils and crops, enhancing crop production and crop quality and reducing micronutrients malnutrition in animals and human being^15^.

The results of continuous assessment of the status of available S and micronutrients and their deficiencies in different cultivated soils help the policy makers and fertilizer industries for planning production and supply of fertilizers having S and micronutrients to districts and states for effective management of these nutrients^11,16^. The geostatistical tools are useful for preparation of S and micronutrients deficiency maps of different areas^17,18,19^. The deficiency maps are helpful in preparing strategies for efficient S and micronutrients management with due emphasis on right nutrient, amount, form and place of application. This helps in better crop production, crop produce quality and soil health^1,6,20,21^.

The information pertaining to state-wise status and deficiency of available S and micronutrients in different states of India is limited, although there are some scattered reports on this aspect. It was hypothesized that there is a wide variability of status and deficiency of available S and micronutrients in cultivated soils of different states of India. The present study was, therefore, carried out to assess the concentrations of available S and micronutrients and levels and distribution of their deficiencies in cultivated soils of various states of India. The results obtained from the study could be highly useful for effective S and micronutrients management in different soil–crop situations of various states of the country.

## Materials and methods

### Study area

The study location viz., India is located at 8° 4′ to 37° 6′ N latitude and 68° 7′ to 97° 25′ E longitude and surrounded by the Indian Ocean (on the south), the Arabian Sea (on the southwest), and the Bay of Bengal (on the southeast). It shares its land borders with Pakistan, China, Bhutan, Bangladesh, Myanmar and Nepal. India is the bulk of Indian subcontinent lying on Indian tectonic plate which is a portion of Indo-Australian plate. It comprises of 28 states and 8 union territories. Soils of the country are alluvial, black, red and yellow, lateritic, arid, forest and mountainous, and are formed by deposition of sediments of rivers. Soils belong to mainly Inceptisols, Alfisols, Entisols, Vertisols, Mollisols, Aridisols, Ultisols and Oxisols orders with sandy to clayey in texture^22^. The climate of the country is influenced by the Himalayas and the Thar desert. India has arid, semi-arid, tropical wet, tropical wet-dry, humid sub-tropical and alpine climatic zones with mean temperature of < 20.0 to > 27.5 °C prevailing across the country. The country is divided into 20 agro-ecological regions (AER) with different climate parameters, soils and cultivated crops^23^ (Supplementary Tables S1, S2). The mean annual rainfall varies from < 150 to 3000 mm in different AERs. It receives uneven and erratic rainfall during June to September months. It rains heavily in north-eastern and Western Ghats regions and Kerala state of the country. Whereas, south-eastern parts and Indo-Gangetic plain receives moderate rainfall. Scanty rainfall is obtained in the western parts of Gujarat, Rajasthan, Punjab and Haryana.

### Soil sampling and analysis

A total of 2,42,827 soil samples from the surface (0–15 cm depth) soil layers were collected from agricultural land holdings of 615 districts lying in 28 states of India, under the tutelage of All India Coordinated Research Project on Micro and Secondary Nutrients and Pollutant Elements in Soils and Plants, by following stratified random sampling procedure^24^, during April to June months of 2012–2018. Soil samples were collected using a hand-held auger made up of stainless-steel. The geographical coordinates of each sampling point were recorded using Global Positioning System. Each composite soil sample was obtained from 3 to 4 subsamples collected from a small land holding (< 1 ha), 6–7 subsamples collected from a medium land holding (1–3 ha) and 9–10 subsamples collected from a large land holding (> 3 ha), of each district. The collected samples were air dried, processed, ground to pass through a 2 mm size sieve and stored in plastic bottles for analysis.

The analysis of soil samples was carried out for available S using calcium chloride solution (0.15%) as extractant^25^ and spectrophotometer (Make (model): Shimadzu (UV-1800)). Available Zn, Fe, Cu and Mn in soil samples were determined after extracting the samples with diethylene triamine penta acetic acid extractant^26^. The estimation of Zn, Fe, Cu and Mn in clear extract was carried out utilizing atomic absorption spectrophotometer (Make (model): Varian (AA240FS)). Available B was estimated after extracting the samples with hot water^27^ and estimating the colour intensity developed by adding azomethine-H solution using a spectrophotometer (Make (model): Shimadzu (UV-1800)).

### Statistical analysis

The dataset related to available S, Zn, B, Fe, Cu and Mn in soil samples were subjected to statistical analysis for obtaining descriptive statistics viz., minimum, maximum, mean, standard deviation (SD), coefficient of variation (CV), skewness and kurtosis, using SAS 9.2 software package^28^. The frequency distribution of soil samples having available S and micronutrients in different concentrations and distribution of single- and multi-nutrients deficiencies were estimated using data analysis programme of Microsoft-excel. The concentrations of S and micronutrients in soil samples were categorized as acute deficient, deficient, latent deficient, marginally sufficient, adequate and high as per the generalized classification adopted for Indian soils^13^ (Table 1). The distribution maps for two and multi-nutrients deficiencies were prepared using ArcGIS software (version 10.5.1) (Environmental Systems Research Institute, Redlands, California) for power BI, in order to have better data visualization and proper understanding of distribution of nutrient deficiencies in different parts of India. The nutrient deficiencies (% of the soil samples), district boundary, state boundary and AER boundary were used as different layers of ArcGIS mapping. Two kriged distribution maps of Zn + B and S + Zn + B deficiency (considering deficient (acute deficient + deficient + latent deficient), marginal (marginally sufficient) and high (adequate + high) status) were prepared using ArcGIS software (version 10.5.1) (Environmental Systems Research Institute, Redlands, California).

**Table 1:** Critical limits of available S and micronutrients for agricultural soils of India.

Nutrients	Acute deficient	Deficient	Latent deficient	Marginal sufficient	Adequate	High
Available S (mg kg^−1^)	≤ 7.50	> 7.50–≤ 15.0	> 15.0–≤ 22.5	> 22.5–≤ 30.0	> 30.0–≤ 40.0	> 40.0
Available Zn (mg kg^−1^)	≤ 0.30	> 0.30–≤ 0.60	> 0.60–≤ 0.90	> 0.90–≤ 1.20	> 1.20–≤ 1.80	> 1.80
Available B (mg kg^−1^)	≤ 0.20	> 0.20–≤ 0.50	> 0.50–≤ 0.70	> 0.70–≤ 0.90	> 0.90–≤ 1.10	> 1.10
Available Fe (mg kg^−1^)	≤ 2.50	> 2.50–≤ 4.50	> 4.50–≤ 6.50	> 6.50–≤ 8.50	> 8.50–≤ 10.5	> 10.5
Available Cu (mg kg^−1^)	≤ 0.20	> 0.20–≤ 0.40	> 0.40–≤ 0.60	> 0.60–≤ 0.80	> 0.80–≤ 1.00	> 1.00
Available Mn (mg kg^−1^)	≤ 1.00	> 1.00–≤ 3.00	> 3.00–≤ 5.00	> 5.00–≤ 7.00	> 7.00–≤ 9.00	> 9.00

### Consent to participate

The consent of all the participants of the study was obtained.

### Consent for publication

The consent for publication was obtained from all the participants of the study.

## Results

### Status of available S and micronutrients

The values of available S and micronutrients varied widely (Supplementary Table S3). The mean concentration (mean ± SD) was 27.0 ± 29.9 mg kg^−1^ for available S, 1.40 ± 1.60 mg kg^−1^ for available Zn, 1.40 ± 4.70 mg kg^−1^ for available B, 31.0 ± 52.2 mg kg^−1^ for available Fe, 2.30 ± 3.50 mg kg^−1^ for available Cu, and 17.5 ± 21.4 mg kg^−1^ for available Mn. The CV values of available S and micronutrients varied from 111 to 338%.

### Single nutrient deficiencies of available S and micronutrients

On average, about 11.4, 29.4 and 17.8% of soils were acute deficient, deficient and latent deficient in available S (Table 2). Whereas, 12.1, 11.6 and 17.7% of soils were marginally sufficient, adequate and high, respectively, in available S. Relatively, the higher % of soils in the states namely Haryana (22.9%), Kerala (31.4%), Odisha (29.5%), Rajasthan (33.9%) and West Bengal (28.6%) were acute deficient in available S. More than 60% of soils in the states namely, Assam, Bihar, Goa, Gujarat, Jharkhand, Karnataka, Kerala, Madhya Pradesh, Maharashtra, Odisha, Rajasthan, Uttar Pradesh and West Bengal were deficient (including acute deficient, deficient and latent deficient) in available S.

**Table 2:** State-wise deficiency (% of soil samples) status of available S.

State	Acute deficient	Deficient	Latent deficient	Marginal sufficient	Adequate	High
Andhra Pradesh	7.70	28.4	12.4	16.2	9.20	26.1
Arunachal Pradesh	1.00	5.00	7.20	29.6	56.7	0.50
Assam	6.20	30.2	29.4	22.7	6.30	5.30
Bihar	8.40	36.2	25.5	15.8	7.80	6.30
Chhattisgarh	9.40	27.9	17.8	27.8	11.2	6.00
Goa	0.00	53.2	7.50	5.10	11.0	23.1
Gujarat	17.4	45.1	16.7	10.1	5.10	5.60
Haryana	22.9	17.2	7.50	5.60	5.30	41.5
Himachal Pradesh	0.90	3.60	3.20	10.6	47.4	34.3
Jammu & Kashmir	15.0	12.9	9.80	12.1	17.8	32.6
Jharkhand	17.8	47.3	15.6	8.60	6.80	3.80
Karnataka	16.1	30.5	16.5	17.1	10.3	9.50
Kerala	31.4	18.0	12.5	13.7	9.50	14.9
Madhya Pradesh	14.7	37.7	19.4	13.4	7.10	7.70
Maharashtra	5.20	37.2	23.5	13.0	10.5	10.7
Manipur	12.3	41.7	5.20	5.50	21.7	13.4
Meghalaya	8.10	10.9	40.8	28.9	10.1	1.20
Mizoram	10.8	11.3	27.4	41.2	9.30	0.00
Nagaland	5.60	11.4	17.5	50.2	12.3	3.00
Odisha	29.5	22.8	14.9	12.3	9.30	11.2
Punjab	15.9	20.1	14.2	15.7	13.0	21.1
Rajasthan	33.9	27.3	17.0	12.0	4.90	4.80
Tamil Nadu	3.20	13.3	13.3	16.5	15.3	38.5
Telangana	11.1	31.5	17.2	15.3	12.3	12.6
Tripura	14.6	16.3	6.20	18.5	38.8	5.60
Uttar Pradesh	11.4	29.4	20.8	19.2	12.8	6.50
Uttarakhand	12.8	25.9	13.7	13.2	9.10	25.3
West Bengal	28.6	32.9	17.0	12.3	5.10	4.00
India	11.4	29.4	17.8	12.1	11.6	17.7

Averagely, about 7.90, 28.6 and 14.7% of soils were acute deficient, deficient and latent deficient in available Zn (Table 3). Whereas, 12.7, 14.8 and 21.3% of soils were marginally sufficient, adequate and high, respectively, in available Zn. Relatively, the higher % of soils in the states namely Goa (23.5%), Karnataka (11.4%), Madhya Pradesh (20.3%), Rajasthan (22.6%), Odisha (13.5%), Bihar (9.10%) and Maharashtra (9.90%) were acute deficient in available Zn. More than 50% of soils in the states namely, Andhra Pradesh, Assam, Bihar, Chhattisgarh, Goa, Gujarat, Karnataka, Madhya Pradesh, Maharashtra, Odisha, Rajasthan, Telangana and Uttar Pradesh were deficient (including acute deficient, deficient and latent deficient) in available Zn.

**Table 3:** State-wise deficiency (% of soil samples) status of available Zn.

State	Acute deficient	Deficient	Latent deficient	Marginal sufficient	Adequate	High
Andhra Pradesh	3.40	27.6	19.8	14.3	16.9	17.9
Arunachal Pradesh	0.30	6.10	4.80	7.90	26.2	54.8
Assam	3.00	39.6	18.6	13.8	11.4	13.6
Bihar	9.10	29.4	14.5	14.0	15.2	17.7
Chhattisgarh	6.60	30.5	18.5	17.8	15.0	11.7
Goa	23.5	35.0	11.9	11.5	15.0	3.10
Gujarat	7.70	36.6	16.3	14.5	13.2	11.8
Haryana	2.10	18.4	11.0	14.7	20.7	33.1
Himachal Pradesh	1.20	8.30	5.80	7.10	20.7	56.9
Jammu & Kashmir	4.40	24.8	12.7	14.3	18.4	25.3
Jharkhand	2.20	19.9	17.8	19.5	20.4	20.2
Karnataka	11.4	27.0	12.1	10.9	13.4	25.2
Kerala	3.80	5.90	4.70	7.10	12.2	66.3
Madhya Pradesh	20.3	46.7	15.9	7.30	5.70	4.20
Maharashtra	9.90	35.7	13.0	11.5	14.8	15.1
Manipur	5.70	19.9	12.5	17.2	23.5	21.3
Meghalaya	0.20	5.60	6.80	8.90	18.9	59.6
Mizoram	0.00	3.50	5.10	6.90	29.4	55.1
Nagaland	0.10	6.60	7.60	7.40	25.2	53.2
Odisha	13.5	27.4	13.7	13.9	14.7	16.9
Punjab	4.20	19.0	12.1	12.6	18.8	33.4
Rajasthan	22.6	41.7	15.6	8.70	6.00	5.30
Tamil Nadu	7.50	23.1	14.4	15.4	17.5	22.0
Telangana	5.80	32.2	16.8	19.3	14.1	11.9
Tripura	0.60	3.90	0.60	2.80	19.7	72.5
Uttar Pradesh	4.00	34.6	22.0	17.3	15.0	7.20
Uttarakhand	1.60	9.70	7.50	11.1	18.9	51.2
West Bengal	2.90	14.3	13.6	18.7	26.2	24.2
India	7.90	28.6	14.7	12.7	14.8	21.3

On average, about 4.00, 19.2 and 21.5% of soils were acute deficient, deficient and latent deficient in available B (Table 4). Whereas, 12.1, 11.4 and 31.7% of soils were marginally sufficient, adequate and high, respectively, in available B. Relatively, the higher % of soils in the states namely Gujarat (18.7%), Jammu & Kashmir (12.1%), Kerala (30.5), Odisha (18.2%), and West Bengal (13.1%) were acute deficient in available B. More than 60% of soils in the states namely Arunachal Pradesh, Assam, Bihar, Gujarat, Himachal Pradesh, Jammu & Kashmir, Jharkhand, Kerala, Maharashtra, Manipur, Meghalaya, Mizoram, Nagaland, Odisha and West Bengal were deficient (including acute deficient, deficient and latent deficient) in available B.

**Table 4:** State-wise deficiency (% of soil samples) status of available B.

State	Acute deficient	Deficient	Latent deficient	Marginal sufficient	Adequate	High
Andhra Pradesh	0.60	10.5	16.4	24.9	17.0	30.6
Arunachal Pradesh	1.20	37.9	50.1	6.50	2.60	1.60
Assam	8.10	38.4	29.7	12.7	2.80	8.4
Bihar	6.80	32.2	24.4	16.4	9.70	10.5
Chhattisgarh	3.40	19.7	11.8	11.7	9.40	43.9
Goa	0.00	12.9	29.1	13.7	12.9	31.3
Gujarat	18.7	38.4	12.9	8.70	5.40	15.9
Haryana	0.20	3.30	5.60	7.9	10.5	72.4
Himachal Pradesh	3.60	28.9	44.8	13.5	5.10	4.10
Jammu & Kashmir	12.1	24.0	23.9	12.9	11.8	15.3
Jharkhand	8.10	41.8	25.0	11.6	6.30	7.20
Karnataka	2.20	33.7	21.5	10.5	6.10	26.0
Kerala	30.5	25.0	12.3	7.80	6.00	18.4
Madhya Pradesh	0.40	5.70	7.60	8.20	6.30	71.8
Maharashtra	3.40	28.3	40.3	13.6	6.00	8.40
Manipur	8.90	43.4	20.5	13.0	7.40	6.80
Meghalaya	1.70	44.3	31.3	9.80	6.10	6.70
Mizoram	2.00	33.0	38.7	19.5	5.10	1.80
Nagaland	0.10	51.3	33.8	6.00	5.30	3.60
Odisha	18.2	35.8	18.2	10.0	5.50	12.3
Punjab	1.20	14.2	17.1	14.7	12.1	40.7
Rajasthan	1.30	6.10	9.90	13.7	11.0	58.0
Tamil Nadu	3.80	19.8	13.3	11.3	8.40	43.4
Telangana	1.80	30.3	27.3	16.1	9.40	15.0
Tripura	1.70	15.7	30.9	27.5	16.3	7.90
Uttar Pradesh	1.00	19.5	19.3	16.2	12.0	32.0
Uttarakhand	6.10	18.0	10.3	7.80	8.80	49.0
West Bengal	13.1	29.4	22.2	12.6	7.80	14.9
India	4.00	19.2	21.5	12.1	11.4	31.7

Averagely, about 3.80, 9.00 and 6.40% of soils were acute deficient, deficient and latent deficient in available Fe (Table 5). Whereas, 9.60, 11.3 and 59.9% of soils were marginally sufficient, adequate and high, respectively, in available Fe. Relatively, the higher % of soils in the states namely Haryana (8.20%), Karnataka (8.90%), Maharashtra (8.50), Rajasthan (5.70%), Tamil Nadu (6.00%), Telangana (5.00%) and Uttar Pradesh (5.40%) were acute deficient in available Fe. More than 25% of soils in the states namely, Andhra Pradesh, Goa, Gujarat, Haryana, Karnataka, Maharashtra, Rajasthan, Tamil Nadu and Telangana were deficient (including acute deficient, deficient and latent deficient) in available Fe.

**Table 5:** State-wise deficiency (% of soil samples) status of available Fe.

State	Acute deficient	Deficient	Latent deficient	Marginal sufficient	Adequate	High
Andhra Pradesh	3.80	11.9	12.1	11.8	7.70	52.6
Arunachal Pradesh	0.00	0.40	11.2	17.0	12.7	58.7
Assam	0.10	0.10	0.00	0.00	0.30	99.5
Bihar	1.60	5.30	4.00	5.60	5.20	78.3
Chhattisgarh	1.90	5.00	3.90	4.80	4.00	80.4
Goa	0.00	15.4	20.1	12.2	7.60	44.6
Gujarat	2.80	20.7	20.2	16.4	9.60	30.4
Haryana	8.20	15.9	9.60	10.8	8.40	47.1
Himachal Pradesh	0.70	2.00	5.60	10.3	10.9	70.5
Jammu & Kashmir	1.20	2.00	3.60	4.50	4.70	84.1
Jharkhand	0.00	0.10	0.00	0.20	0.10	99.6
Karnataka	8.90	11.7	5.80	7.00	6.70	59.9
Kerala	1.30	1.60	1.60	1.70	1.70	92.2
Madhya Pradesh	2.70	7.60	10.1	12.6	11.7	55.3
Maharashtra	8.50	17.2	10.7	10.4	8.90	44.3
Manipur	0.10	1.20	5.70	7.30	6.10	79.5
Meghalaya	0.10	1.60	8.10	10.8	9.10	70.2
Mizoram	0.00	0.40	7.70	7.50	8.60	75.7
Nagaland	0.60	2.10	7.90	10.8	9.90	68.7
Odisha	1.60	4.30	2.60	3.10	2.40	86.0
Punjab	3.90	10.9	7.10	8.10	7.80	62.2
Rajasthan	5.70	34.8	25.1	11.2	4.60	18.5
Tamil Nadu	6.00	11.1	8.40	9.40	8.00	57.1
Telangana	5.00	10.4	9.80	11.5	10.2	53.1
Tripura	0.60	0.60	2.20	1.70	7.30	87.6
Uttar Pradesh	5.40	12.4	13.5	13.6	10.7	44.4
Uttarakhand	0.30	1.70	2.60	5.70	6.30	83.6
West Bengal	0.00	0.00	0.00	0.10	0.00	99.9
India	3.80	9.00	6.40	9.60	11.3	59.9

Averagely, about 2.10, 2.10 and 7.20% of soils were acute deficient, deficient and latent deficient in available Cu (Table 6). Whereas, 10.8, 10.2 and 67.6% of soils were marginally sufficient, adequate and high, respectively, in available Cu. Relatively, the higher % of soils in the states namely Haryana (6.20%), Odisha (5.70%). Punjab (4.70), Rajasthan (9.10%), and Uttar Pradesh (3.10%) were acute deficient in available Cu. More than 25% of soils in the states namely, Goa, Haryana, Punjab, Rajasthan, and Uttar Pradesh were deficient (including acute deficient, deficient and latent deficient) in available Cu.

**Table 6:** State-wise deficiency (% of soil samples) status of available Cu.

State	Acute deficient	Deficient	Latent deficient	Marginal sufficient	Adequate	High
Andhra Pradesh	1.30	5.30	9.00	11.1	10.1	63.2
Arunachal Pradesh	1.40	3.90	11.8	19.8	15.8	47.2
Assam	2.20	8.50	5.30	4.30	4.10	75.7
Bihar	0.70	1.50	1.60	2.30	3.20	90.7
Chhattisgarh	2.60	4.50	4.70	5.30	5.40	77.4
Goa	3.10	41.2	22.1	14.4	6.30	12.9
Gujarat	0.40	2.80	6.40	7.90	9.00	73.6
Haryana	6.20	13.8	10.7	9.40	9.70	50.2
Himachal Pradesh	1.40	4.80	8.50	15.0	13.4	56.8
Jammu & Kashmir	0.80	3.40	3.90	6.60	6.00	79.3
Jharkhand	0.50	1.70	4.60	6.70	6.40	80.2
Karnataka	2.10	4.80	7.90	8.60	9.80	66.7
Kerala	2.60	3.60	3.80	4.30	4.60	81.1
Madhya Pradesh	0.50	3.30	5.80	7.00	8.10	75.4
Maharashtra	0.10	1.10	2.20	3.40	4.00	89.0
Manipur	1.40	5.70	8.60	14.0	11.1	59.2
Meghalaya	1.00	5.20	5.40	9.90	12.3	66.3
Mizoram	1.50	6.40	5.10	16.4	14.4	56.2
Nagaland	0.50	9.60	11.6	16.9	11.2	50.1
Odisha	5.70	5.80	4.80	6.00	5.10	72.7
Punjab	4.70	12.3	13.5	13.8	13.8	41.8
Rajasthan	9.10	34.6	22.0	9.80	6.70	17.7
Tamil Nadu	1.40	4.00	5.90	7.80	8.40	72.3
Telangana	1.40	5.80	7.00	7.20	7.40	71.2
Tripura	1.70	1.10	0.00	7.30	9.60	80.3
Uttar Pradesh	3.10	13.2	12.1	9.70	8.30	53.6
Uttarakhand	1.60	6.10	10.1	11.8	11.0	59.4
West Bengal	1.50	2.70	2.10	2.60	2.80	88.5
India	2.10	2.10	7.20	10.8	10.2	67.6

On average, about 1.10, 6.00 and 10.3% of soils were acute deficient, deficient and latent deficient in available Mn (Table 7). Whereas, 13.2, 9.10 and 60.4% of soils were marginally sufficient, adequate and high, respectively, in available Mn. Relatively, the higher % of soils in the states namely Jammu & Kashmir (3.50%), Kerala (3.40%), Punjab (7.80), and Rajasthan (5.80%) were acute deficient in available Mn. More than 20% of soils in the states namely, Bihar, Goa, Haryana, Jammu & Kashmir, Kerala, Nagaland, Punjab, Rajasthan, Tamil Nadu and Uttar Pradesh were deficient (including acute deficient, deficient and latent deficient) in available Mn.

**Table 7:** State-wise deficiency (% of soil samples) status of available Mn.

State	Acute deficient	Deficient	Latent deficient	Marginal sufficient	Adequate	High
Andhra Pradesh	0.60	4.00	8.50	7.80	7.20	71.9
Arunachal Pradesh	0.10	5.70	9.00	21.6	18.5	45.1
Assam	0.10	0.30	3.40	6.60	3.40	86.2
Bihar	0.60	8.70	14.3	15.1	12.0	49.4
Chhattisgarh	0.80	3.50	6.40	5.60	4.70	79.0
Goa	0.00	15.0	5.90	9.90	15.7	53.5
Gujarat	0.10	2.20	7.60	10.4	10.3	69.5
Haryana	1.30	7.40	12.8	13.3	12.6	52.7
Himachal Pradesh	0.20	4.50	8.90	16.6	17.4	52.4
Jammu & Kashmir	3.50	18.7	16.7	11.4	9.90	39.7
Jharkhand	0.10	0.30	0.90	1.50	2.20	95.0
Karnataka	1.90	8.10	8.10	7.70	6.90	67.3
Kerala	3.40	9.10	9.10	6.50	5.70	66.3
Madhya Pradesh	0.20	6.10	10.1	10.5	9.90	63.2
Maharashtra	0.10	2.00	5.60	7.30	7.90	77.2
Manipur	0.00	10.3	6.50	13.1	13.4	56.5
Meghalaya	0.10	5.90	10.7	12.7	9.90	60.7
Mizoram	0.00	3.10	4.00	13.3	11.3	68.4
Nagaland	0.30	10.8	9.60	12.2	12.2	54.8
Odisha	0.60	4.20	4.50	4.60	3.90	82.2
Punjab	7.80	21.2	26.3	18.7	10.9	15.2
Rajasthan	5.80	30.5	17.3	14.6	10.8	21.0
Tamil Nadu	1.70	12.6	12.7	9.50	8.10	55.4
Telangana	0.00	8.40	9.20	8.60	7.50	66.2
Tripura	0.60	0.60	1.10	6.70	5.60	85.4
Uttar Pradesh	0.50	10.2	15.4	15.4	9.20	49.3
Uttarakhand	0.20	4.80	5.60	7.40	7.70	74.3
West Bengal	0.30	1.60	2.50	3.40	3.50	88.7
India	1.10	6.00	10.3	13.2	9.10	60.4

### Multi-nutrients deficiencies of available S and micronutrients

**Figure 1:**
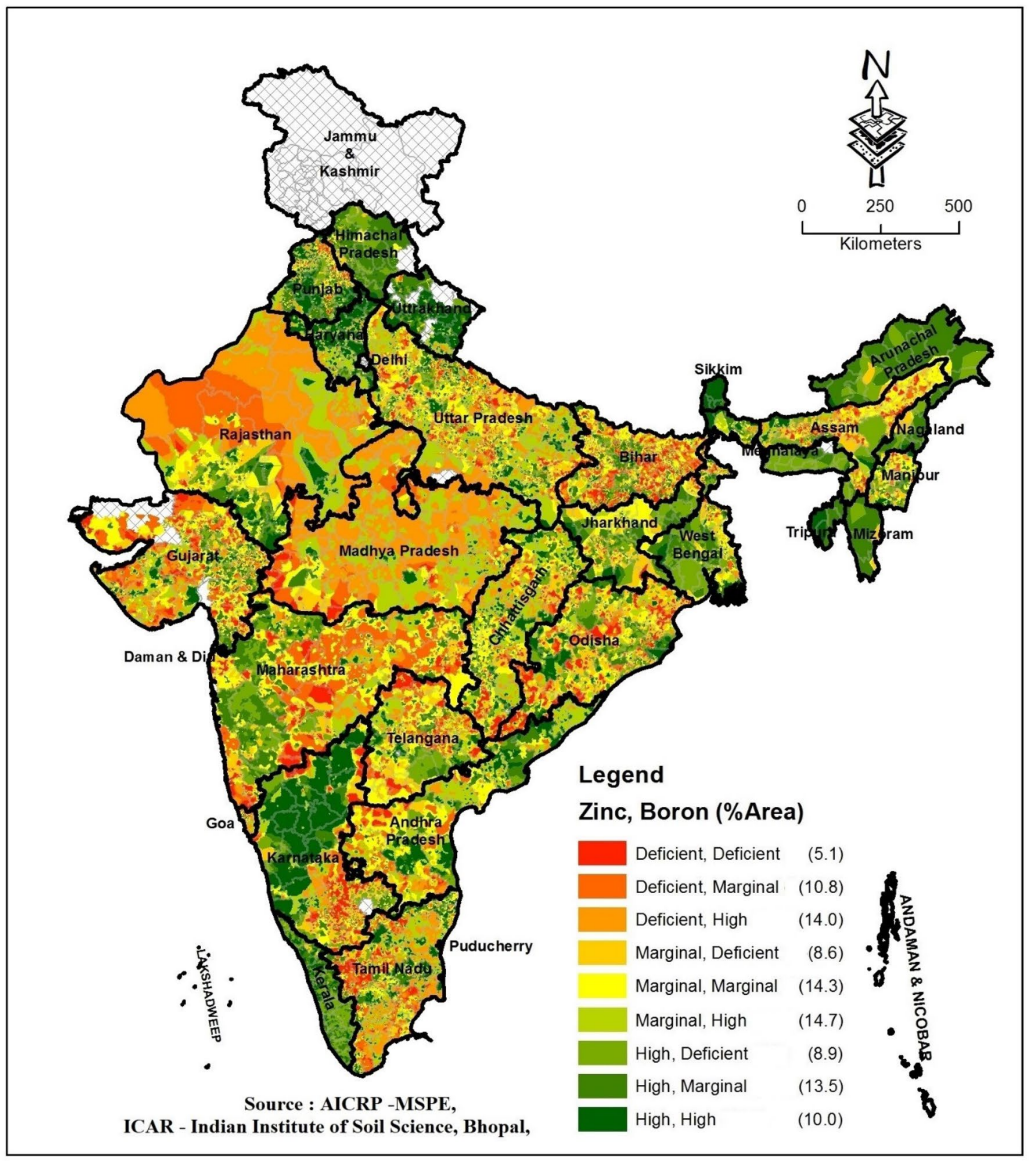
Spatial distribution of Zn + B deficiency in different states of India (The figure has been prepared using ArcGIS software (version 10.5.1), Environmental Systems Research Institute, Redlands, California).

The mean deficiency of 2 or > 2-nutrients deficiency of available S and micronutrients varied from 0.10% (Zn + Fe + Cu + Mn + B) to 9.30% (S + Zn) (Table 8). The deficiency of S + Zn was predominantly prevalent in different districts of Bihar, Gujarat, Karnataka, Madhya Pradesh, Odisha, Rajasthan, Uttar Pradesh and Maharashtra (Supplementary Figure S1). More than 20% of soils in 9 districts of Gujarat, 25 districts of Madhya Pradesh, 8 districts of Odisha and 10 districts of Rajasthan were deficient in S + Zn. The deficiency of Zn + B varied from 0.60 to 20.3% of soils in different states with mean value of 8.70%. It’s prevalence in states like Bihar, Karnataka, Odisha and Tamil Nadu was higher (Fig. 1, Supplementary Figure S2). More than 20% of the sampled sites in 16 districts of Bihar, 5 districts each of Karnataka and Telangana, 13 districts of Odisha and 7 districts of Tamil Nadu were deficient in Zn + B. The deficiency of S + B varied widely in different states with mean value of 7.00%. Relatively higher % of the soils in Jharkhand, Karnataka, Kerala, Manipur, Odisha, Uttarakhand and West Bengal state were deficient in S + B (Supplementary Figure S3). More than 20% of soils in 6 districts each of Jharkhand and Manipur, 9 districts each of Karnataka and Kerala, 14 districts of Odisha and 5 districts of Telangana were deficient in S + B. On average, 5.80% of soils were deficient in Zn + Fe. Relatively higher % of soils of Gujarat, Karnataka, Maharashtra and Rajasthan were deficient in Zn + Fe (Supplementary Figure S4). More than 20% of the sampled sites in 9 districts of Gujarat, 5 districts each of Madhya Pradesh and Maharashtra and 7 districts of Rajasthan had Zn + Fe deficiency. The higher % of soils of Goa, Jammu & Kashmir and Rajasthan were deficient in Zn + Mn with a national average of 3.4% (Supplementary Figure S5). The deficiency of S + Fe, Zn + Cu and Fe + B were recorded in 3.30, 2.80 and 2.70% of the sampled sites, respectively and were scattered across the country (Supplementary Figure S6–S8). The deficiency S + Fe was higher in Gujarat, Haryana, Rajasthan and Uttar Pradesh.

**Table 8:** Per cent of the soils deficient in multi-nutrients in different states of India.

State	S + Zn	Zn + B	S + B	Zn + Fe	Zn + Mn	S + Fe	Zn + Cu	Fe + B	S + Zn + B	S + Zn + Fe	Zn + Fe + B	Zn + Fe + Cu + Mn	Zn + Fe + Cu + Mn + B
Andhra Pradesh	6.23	2.06	2.59	6.90	0.60	4.65	0.60	1.78	0.73	1.88	0.62	0.01	0.00
Arunachal Pradesh	0.43	2.41	2.91	0.00	0.50	0.00	0.30	0.00	0.28	0.00	0.00	0.00	0.00
Assam	5.43	7.88	6.78	0.00	0.00	0.00	0.90	0.14	1.95	0.00	0.00	0.00	0.00
Bihar	12.9	19.6	9.70	5.26	4.80	2.26	2.20	7.90	5.94	1.55	3.67	0.23	0.19
Chhattisgarh	11.2	8.87	7.79	3.04	0.90	2.70	0.90	0.87	3.86	1.57	0.63	0.02	0.00
Goa	17.6	6.32	9.26	2.21	10.30	0.59	2.10	0.59	5.15	0.44	0.29	0.00	0.00
Gujarat	15.5	7.29	7.98	11.7	0.30	12.7	0.30	4.35	3.23	5.81	1.94	0.01	0.00
Haryana	6.54	0.75	1.39	6.59	2.50	8.70	3.40	1.10	0.37	3.02	0.44	0.34	0.09
Himachal Pradesh	0.15	3.08	0.97	0.22	0.80	0.25	0.30	0.50	0.14	0.03	0.09	0.00	0.00
Jammu & Kashmir	4.44	0.00	0.00	0.54	7.04	0.43	0.19	0.00	0.00	0.20	0.00	0.07	0.00
Jharkhand	7.38	8.40	25.9	0.00	0.20	0.00	0.10	0.00	4.40	0.00	0.00	0.00	0.00
Karnataka	12.2	15.3	13.9	9.24	1.20	3.88	1.10	6.84	6.12	1.95	3.31	0.02	0.01
Kerala	2.61	3.93	21.3	0.97	0.70	1.23	0.50	2.10	1.37	0.38	0.71	0.14	0.14
Madhya Pradesh	18.8	2.27	1.66	5.83	1.50	2.70	0.40	0.60	0.98	1.87	0.39	0.00	0.00
Maharashtra	10.0	5.19	3.52	9.82	1.80	5.38	0.10	5.23	1.20	3.09	1.48	0.01	0.00
Manipur	5.17	9.41	17.4	0.87	2.40	0.07	0.60	0.93	2.52	0.00	0.43	0.00	0.00
Meghalaya	0.55	1.31	3.37	0.07	0.30	0.76	0.10	0.62	0.14	0.00	0.07	0.00	0.00
Mizoram	0.22	1.11	3.76	0.00	0.00	0.22	0.20	0.22	0.00	0.00	0.00	0.00	0.00
Nagaland	0.50	2.19	0.80	0.40	0.30	0.60	0.10	0.60	0.00	0.00	0.30	0.00	0.00
Odisha	18.0	20.3	25.3	2.53	1.30	1.67	2.50	2.25	11.4	1.01	1.04	0.01	0.00
Punjab	6.15	2.83	4.04	4.64	7.00	4.90	2.70	1.82	1.01	1.70	0.68	0.48	0.15
Rajasthan	29.9	0.57	1.16	23.3	22.5	16.9	7.80	0.06	0.30	11.8	0.02	2.03	0.00
Tamil Nadu	5.81	13.1	2.45	7.71	5.30	0.88	9.10	2.00	1.68	0.71	1.38	0.66	0.10
Telangana	6.96	8.92	9.60	4.68	0.80	4.48	0.60	4.10	2.26	1.22	1.30	0.00	0.00
Tripura	0.00	0.56	0.00	0.00	0.00	0.00	0.00	0.00	0.00	0.00	0.00	0.00	0.00
Uttar Pradesh	8.81	5.52	6.57	5.25	4.80	5.71	1.20	2.75	2.17	2.36	0.97	0.32	0.19
Uttarakhand	1.87	1.26	11.9	0.44	1.10	0.30	0.70	0.49	0.55	0.12	0.09	0.09	0.00
West Bengal	7.97	4.17	19.44	0.00	0.40	0.00	0.50	0.02	2.30	0.00	0.00	0.00	0.00
India	9.30	8.70	7.00	5.80	3.40	3.30	2.80	2.70	2.60	1.70	1.20	0.30	0.10

*S* available sulphur, *Zn* available zinc, *B* available boron, *Fe* available iron, *Cu* available copper, *Mn* available manganese.

**Figure 2:**
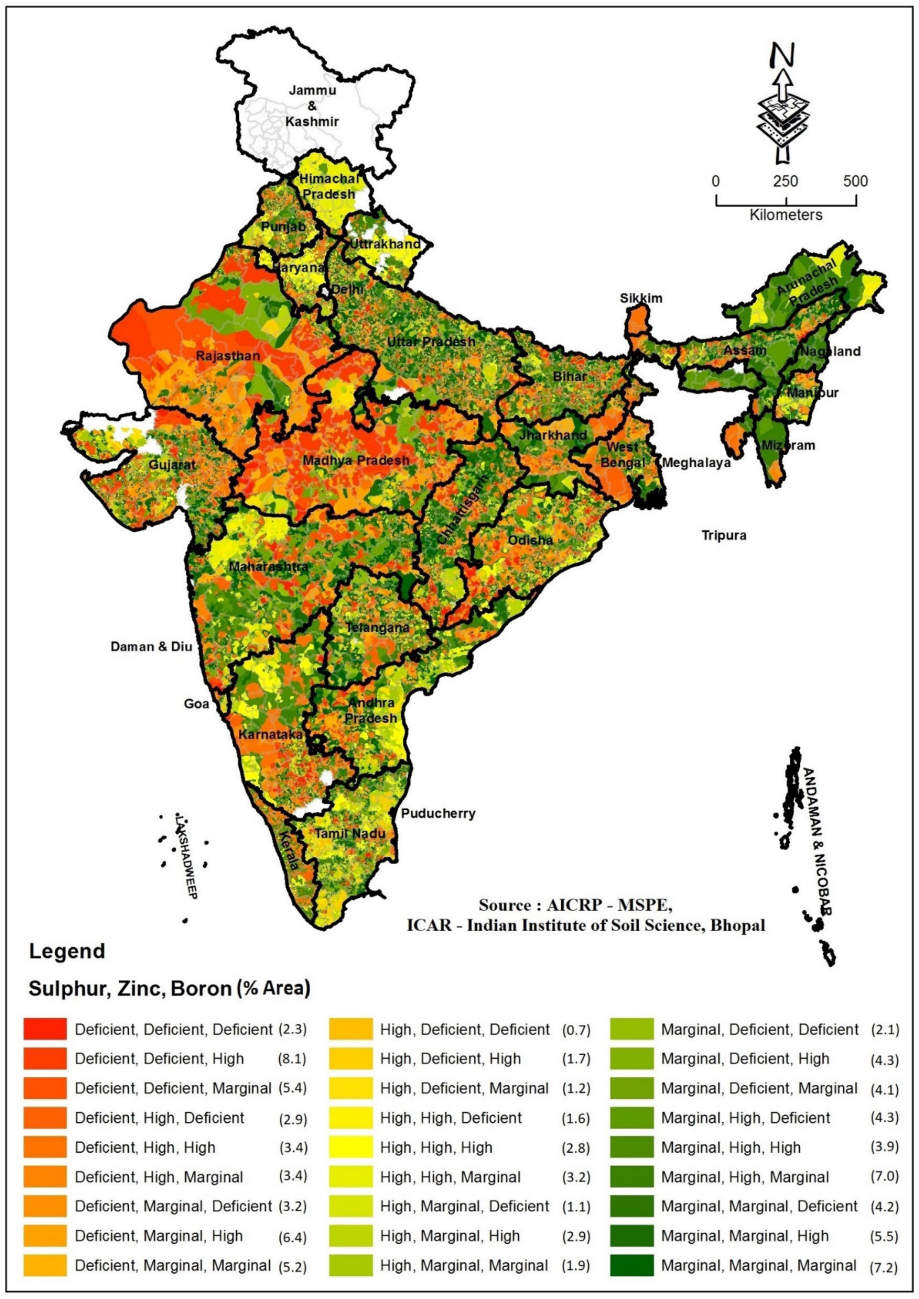
Spatial distribution of S + Zn + B deficiency in different states of India (The figure has been prepared using ArcGIS software (version 10.5.1), Environmental Systems Research Institute, Redlands, California).

The deficiency of 3-nutrients namely, S + Zn + B, S + Zn + Fe and Zn + Fe + B were recorded in 2.60, 1.70 and 1.20% of soils, respectively. Relatively, the higher % of soils in Bihar, Goa, Karnataka and Odisha were deficient in S + Zn + B (Fig. 2). S + Zn + Fe deficiency was more in soils of Gujarat, Haryana, Maharashtra and Rajasthan (Supplementary Figure S9, S10). The higher % of the sampled sites in Bihar and Karnataka were deficient in Zn + Fe + B (Supplementary Figure S11). More than 20% of soils in 1 district each of Bihar, Chhattisgarh, Karnataka, Madhya Pradesh and 5 districts of Odisha were deficient in S + Zn + B. More than 20% of soils in 1 district each of Haryana, Karnataka, Maharashtra, and Uttar Pradesh and 2 districts each of Madhya Pradesh and Rajasthan were deficient in S + Zn + Fe. More than 3-nutrients deficiencies like Zn + Fe + Cu + Mn and Zn + Fe + Cu + Mn + B were very less and recorded in only 0.30 and 0.10% of soils, respectively. Less than 5% of the sampled sites in 13 districts of Bihar, 7 districts each of Punjab and Uttar Pradesh and 16 districts of Tamil Nadu were deficient in Zn + Fe + Cu + Mn. Whereas, < 5% of the sampled sites in 13 districts of Bihar, 4 districts of Punjab and 10 districts of Tamil Nadu were deficient in Zn + Fe + Cu + Mn + B.

## Discussion

### Status of available S and micronutrients

The concentration of available S in soils of different states of India varied widely (from 0.02 to 822 mg kg^−1^). Scherer^29^ also recorded wide differences in the concentrations of available S soils of the world. The concentration of Zn (from 0.01 to 59.8 mg kg^−1^), B (from 0.01 to 109 mg kg^−1^), Fe (from 0.01 to 964 mg kg^−1^), Cu (from 0.01 to 99.2 mg kg^−1^) and Mn (from 0.01 to 483 mg kg^−1^) in soils of the country varied widely. Similarly, Mathew et al.^30^ recorded wide variations in concentration of available Zn, Fe, Mn, B and Cu in cultivated soils of Tanzania.

The mean concentrations of available Zn 2.30 ± 1.30 mg kg^−1^, available Mn 12.2 ± 14.4 mg kg^−1^, available Cu 1.10 ± 0.80 mg kg^−1^ and available Fe 66.6 ± 56.0 mg kg^−1^ were reported by Silva et al.^31^, in sugarcane cultivated soils of Brazil. The factors like soil types, nature of crop plants and adoption of various soil–crop management practices influence the concentrations of available S and micronutrients in soils^12,29,32,33^. The available S concentration in soils is also influenced by occurrences of plant residues, organic matter and various salts containing S^34^. The extent of rock weathering in soil decides the concentration of available micronutrients and S in soils^12^. The lowest concentrations of available S and micronutrients prevail in the tropical soils with high levels of weathering. The availability of micronutrients in soils is primarily affected by soil parameter namely soil organic matter, soil pH, clay content, cation exchange capacity, biological activity and redox potential. The nature of crop plants, rooting pattern, root secretions and associations of plants and microbes also affect micronutrient availability in soil and plant uptake.

### Single nutrient deficiencies of available S and micronutrients

On an average, the concentration of available S in about 11.4, 29.4 and 17.8% of soils were acute deficient, deficient, and latently deficient range, respectively (Table 2). There were differences in deficiency levels of available S in the districts of the states. Several districts in the states like Madhya Pradesh, Gujarat, Odisha, Rajasthan and Manipur had deficiency in available S in > 50% of soils. This is mainly ascribed to variations in cultivation of S-loving crops and less or no addition of S containing fertilizers. The deficiency of available S could be efficiently alleviated by adopting site-specific S manipulation strategies in various soil–crop contexts. In parallel to our findings, several scholars recorded S deficiency in different soil–crop contexts and responses of various crops to different S doses in various states of India^13,35–37^. On average, the extent of deficiency (acute deficient + deficient + latent deficient) was 51.2% for available Zn, 44.7% for available B, 19.2% for available Fe, 11.4% for available Cu and 17.4% for available Mn. In parallel to our results, there were reports of soil micronutrients deficiency in different countries of the world^9^. The districts of various states had different levels of micronutrients deficiency. But the almost all states of India had different levels of Zn deficiency. Most of the soils having low organic carbon content, high soil pH, with coarse textured and calcareousness and under intense cultivation, had Zn deficiency. Similar to Zn deficiency, the deficiency of B at various levels prevailed in soils of various states. However, the lateritic and limed acidic soils, calcareous and leached sandy soils had higher levels of B deficiency. The higher levels of B deficiency in eastern parts of the country are ascribed to alluvial and loess depositions and high levels of leaching in sandy-loam soils. The extent of Fe deficiency was higher in states namely Gujarat, Rajasthan and Maharashtra lying in the western part of the country. Significant % soils of the states like Uttar Pradesh, Bihar, Telangana and Karnataka had also Fe deficiency. Iron deficiency in these soils is mainly due to alkaline soil Ph and moisture deficit situation resulting in transformation of Fe from ferrous (Fe^2+^ soluble) to ferric (Fe^3+^ insoluble) state. The extent of Cu deficiency was the lowest among the studied nutrients. Thirteen districts of Haryana, Assam, Tamil Nadu, Odisha and Rajasthan had Cu deficiency in > 20% of soils. Calcareous, sandy textured and eluviated organic matter rich soils are the causes of Cu deficiency. The soils of the state like Haryana, Bihar, Rajasthan, Tamil Nadu, Uttar Pradesh, Telangana, Punjab and Jammu & Kashmir had higher levels of Mn deficiency. The prime causes of Mn deficiency in these soils are prevailing moisture deficit condition, low total Mn content in soil, coarse textured and calcareous soils. There are reports of responses of different crops to micronutrients addition to various soils of India^19,38,39^, corroborating our findings of micronutrients deficiency in soils of various states. The farmers should go for addition of micronutrients fertilizers based on deficiency levels in soils and nature of crops and their demand, as the crop responses differ with soil–crop contexts.

### Multi-nutrients deficiencies of available S and micronutrients

There were 2 and > 2-nutrients deficiencies of available S and micronutrients in soils different states of India. The extent of these deficiencies followed the order: S + Zn (9.30%) > Zn + B (8.70%) > S + B (7.00%) > Zn + Fe (5.80%) > Zn + Mn (3.40%) > S + Fe (3.30%) > Zn + Cu (2.80%) > Fe + B (2.70%) > S + Zn + B (2.60%) > S + Zn + Fe (1.70%) > Zn + Fe + B (1.20%) > Zn + Fe + Cu + Mn (0.30%) > Zn + Fe + Cu + Mn + B (0.10%) (Table 8). The different states and AERs of the country had various levels of 2 and > 2-nutrients deficiencies of available S and micronutrients. S + Zn deficiency was prevalent in > 15% of soils of Odisha, Rajasthan, Madhya Pradesh and Goa. This was also prevailed in higher extent in soils of AER 5, 8, 9, 10 and 13. The deficiency of Zn + B was prevalent at greater extent in soils of Odisha, Tamil Nadu, Karnataka and Bihar falling in the AER 5, 8, 12 and 13. The soil of the states namely Jharkhand, Odisha, Kerala, West Bengal and Uttarakhand had higher levels of S + B deficiency. The levels of Zn + Fe deficiency were more in Rajasthan, Gujarat, Karnataka and Maharashtra and AER 2, 5, 6 and 8. As discussed earlier, this spatial variations in prevalence of multi-nutrients deficiencies are ascribed to different soils, crops, climates and soil–crop manipulation practices. These multi-nutrients deficiencies could be alleviated by production, distribution and application of S and micronutrients containing customized fertilizers prepared based on prevailing nutrient deficiencies in various AER, states and districts of the country. This will help to a greater extent in maintaining soil health, having sustainable crop production and better quality of crops^40,41^. The information from the study could suitably be used by the different stake holders (policy makers, planners and fertilizer industries) associated with production and distribution of S and micronutrients containing straight and customized fertilizers to various targeted areas of the country. There are responses of different crops to the addition of available customized fertilizers in different parts of India^42–44^. However, there is an urgent necessity for development, distribution and application of S and micronutrients containing straight and customized fertilizers for different areas of the country, based on the current knowledge on S and micronutrients deficiency. It will be useful to a greater extent for alleviating S and micronutrients deficiencies in soils, maintaining soil health, sustainable crop production, increased crop quality and better health of animals and human being.

## Conclusions

The present study highlighted the existence of wide variability in available S and micronutrients status in cultivated soils of various states of India. On average, the extent of deficiencies (considering acute deficient, deficient and latent deficient together) of the studied nutrients in soils of the country followed the order: available S > available Zn > available B > available Fe > available Mn > available Cu. The higher % of soils in Kerala, Odisha and West Bengal were acute deficient in available S and available B. Whereas, the higher % of soils in Rajasthan, Madhya Pradesh, Goa, Odisha and Karnataka were acute deficient in available Zn. The existence of 2 or > 2 nutrients deficiencies of available S and micronutrients is restricted to limited areas of different districts of various states. On average, the levels of deficiencies of S + Zn, Zn + B, S + B and Zn + Fe were higher than the deficiencies of other multi-nutrients. This knowledge could be used for area-specific S and micronutrients management for better crop production and crop quality. Further, district- and state-specific S and micronutrients based customized fertilizers could be developed and distributed based on the information generated from the study. However, there is a need for periodic assessment of available S and micronutrients status in cultivated soils of various states, at 3–5 years interval, as the status of these nutrients change with soil–crop management practices. Further, development of kriged distribution maps of available S and micronutrients (using geostatistical tools) for different districts, states and at country level is needed for preparing site-specific nutrient management strategies. This type of study needs to be carried out in cultivated soils pf other parts of world for effective S and micronutrients needed for substantiable crop production, crop quality and good health of soils, crops, animals and human being.

## Supplementary Information

Supplementary Information.

## Data Availability

The data are available from the corresponding author upon reasonable request.
