# Supplementary material for: Deficiency of phyto-available sulphur, zinc, boron, iron, copper and manganese in soils of India
Source: Sci Rep. 2021 Oct 5;11:19760. doi: 10.1038/s41598-021-99040-2 (PMC8492626; doi:10.1038/s41598-021-99040-2)
Supplement: Supplementary file 1 — Supplementary Information. [file 41598_2021_99040_MOESM1_ESM.docx]

**Supplementary information**

**Deficiency of phyto-available sulphur, zinc, boron, iron, copper and manganese in soils of India**

**Arvind Kumar Shukla^1^, Sanjib Kumar Behera^1, *^, Chandra Prakash^1^, Ajay Tripathi^1^, Ashok Kumar Patra^1^, Brahma Swaroop Dwivedi^2^, Vivek Trivedi^3^, Ch. Srinivasa Rao^4^, Suresh Kumar Chaudhari^5^, Soumitra Das^6^, Anil Kumar Singh^5^**

*^1^ICAR- Indian Institute of Soil Science, Bhopal, 462038, Madhya Pradesh, India*

*^2^ICAR-National Bureau of Soil Survey and Land Use Planning, Nagpur, 440033, Maharashtra, India*

*^3^ICAR-Indian Agricultural Research Institute, New Delhi, 110012, India*

*^4^ICAR-National Academy of Agricultural Research Management, Hyderabad, Telangana 500030, India*

*^5^Indian Council of Agricultural Research, New Delhi, 110012, India*

*^6^International Zinc Association, New Delhi, 110062, India*

Table S1. Different agro-ecological regions of India with climate parameters

| Serial no. of agro-ecological region | Name of agro-ecological region | Climate parameters |
| --- | --- | --- |
| 1 | Cold arid eco-region with shallow skeletal soils | It experiences mild summer, harsh winter and receives mean annual rainfall of < 150 mm. |
| 2 | Hot arid eco-region with desert and saline soils | It experiences hot summer and cool winter and receives mean annual rainfall of < 400 mm. |
| 3 | Hot arid eco-region with red and black soils | It experiences hot and dry summer, mild winter and receives mean annual rainfall of 400-500 mm. |
| 4 | Hot semi-arid eco-region with alluvium derived soils | It experiences hot and dry summer, cool winter and receives mean annual rainfall of 500-1000 mm. |
| 5 | Hot semi-arid eco-region with medium and deep black soils | It experiences hot and wet summer, dry winter and receives mean annual rainfall of 500-1000 mm. |
| 6 | Hot semi-arid eco-region with shallow and medium (dominant) black soils | It experiences hot and humid summer, mild and dry winter and receives mean annual rainfall of 600-1000 mm. |
| 7 | Hot semi-arid eco-region with red and black soils | It experiences hot and dry summer, mild and dry winter and receives mean annual rainfall of 600-1100 mm. |
| 8 | Hot semi-arid eco-region with red loamy soils | It experiences hot and dry summer, mild winter and receives mean annual rainfall of 600-1000 mm. |
| 9 | Hot sub-humid (dry) eco-region with alluvial-derived soils | It experiences hot summer, cool winter and receives mean annual rainfall of 1000-1200 mm. |
| 10 | Hot sub-humid eco-region with red and black soils | It experiences hot summer, mild winter and receives mean annual rainfall of 1000-1500 mm. |
| 11 | Hot sub-humid eco-region with red and yellow soils | It experiences hot summer, cool winter and receives mean annual rainfall of 1200-1600 mm. |
| 12 | Hot sub-humid eco-region with red and lateritic soils | It experiences hot summer, cool winter and receives mean annual rainfall 1000-1600 mm. |
| 13 | Hot sub-humid (moist) eco-region with alluvium-derived soils | It experiences hot and wet summer, cool and dry winter and receives mean annual rainfall of 1400-1800 mm. |
| 14 | Warm sub-humid to humid with inclusion of perhumid eco-region with brown forest and podzoloc soils | It experiences mild summer, cool winter and receives mean annual rainfall of 1000-2000 mm. |
| 15 | Hot sub-humid (moist) to humid (inclusion of perhumid) eco-region with alluvium-derived soils | It experiences hot summer, mild to moderately cool winter and receives mean annual rainfall of 1400-1600 mm. |
| 16 | Warm sub-humid eco-region with brown and red hill soils | It experiences warm summer, cool winter and receives mean annual rainfall of 2000 mm. |
| 17 | Warm perhumid eco-region with red and lateritic soils | It experiences warm summer, cool winter and receives mean annual rainfall of 2000-3000 mm. |
| 18 | Hot sub-humid to semi-arid eco-region with coastal alluvium-derived soils | It experiences semi-arid and sub-humid (moist) climate and receives mean annual rainfall of 900-1100 mm (west coast) and 1200-1600 mm (east coast). |
| 19 | Hot humid perhumid eco-region with red, lateritic and alluvium-derived soils | It experiences hot and humid summer, warm winter and receives mean annual rainfall of >2000 mm. |
| 20 | Hot humid per-humid island eco-region with red loamy and sandy soils | It experiences tropical climate and receives mean annual rainfall of 1600-3000 mm. |

Table S2. Nature of soils and commonly cultivated crops in different agro-ecological regions of India

| Serial no. of agro-ecological region | Nature of soil | Commonly cultivated crops |
| --- | --- | --- |
| 1 | Soils are skeletal, calcareous and alkaline in reaction. | Vegetables, millets, wheat, fodder, pulses and barley. |
| 2 | Soils are sandy, moderately calcareous and alkaline in reaction. | Pearl millet, fodder, pulses, cotton, sugarcane, mustard, wheat and gram. |
| 3 | Soils are shallow, medium red soil with slightly acidic and noncalcareous nature. Soils are also deep, clayey black with slightly alkaline and calcareous nature. | Pearl millet, groundnut, sugarcane, sunflower and cotton. |
| 4 | Soils are deep, loamy and alluvium derived and clayey mixed red and black. | Rice, millets, maize, pulses, berseem, wheat, mustard and sugarcane, jowar, pigeonpea, soybean, lentil and cotton. |
| 5 | Soils are deep loamy to clayey black soil with slightly alkaline and calcareous nature with swell-shrink characters. | Sorghum, pearl millet, pigeonpea, groundnut, soybean, maize, pulses, safflower, sunflower and wheat. |
| 6 | Soils are shallow, loamy skeletal, clayey, calcareous and moderately alkaline in reaction. | Sorghum, pearl millet, pigeonpea, safflower, sunflower, cotton and groundnut. |
| 7 | Soils are red, non-calcareous and neutral in reaction, clayey and calcareous with alkaline in reaction. | Sorghum, pigeonpea, rice, cotton, groundnut, sunflower, safflower, castor. |
| 8 | Soils are non-calcareous and slightly acidic soil, and calcareous and moderately alkaline. | Millets, pulses, groundnut, sorghum and sunflower. |
| 9 | Soils are alluvium derived deep and loamy with neutral in reaction. | Rice, maize, barley, pigeonpea, jute, wheat, lentil, mustard, cotton and sugarcane. |
| 10 | Soils are deep black calcareous soil with slightly alkaline in reaction, moderately deep, clayey red with neutral to slightly acidic in reaction. | Rice, wheat, sorghum, soybean, pigeonpea, gram, cotton and vegetables. |
| 11 | Soils are deep, loamy, non-calcareous, red and yellow with neutral to slightly alkaline in reaction. | Rice, wheat, millets, pigeonpea, green gram and black gram. |
| 12 | Soils are fine loamy to clayey, non-calcareous red soil with slightly to moderately acidic in reaction. | Rice, wheat, pulses and groundnut. |
| 13 | Soils are deep, loamy, calcareous with moderately alkaline in reaction. | Rice, maize, moong, pigeonpea, lentil, wheat, sesamum, groundnut, sugarcane, tobacco, turmeric, chillies, potato and coriander. |
| 14 | Soils are shallow to deep brown forest and podzolic soil, fine loamy and neutral to mildly alkaline in reaction. | Wheat, millet, maize, rice and apple. |
| 15 | Soils are slight to strongly acidic. | Rice, wheat, jute, tea, pineapple, citrus, banana, pulses, oilseed and sugarcane. |
| 16 | Soils are shallow to moderately shallow, loamy brown forest soil with moderately acidic in reaction. | Rice, maize, millet, potato, sweet potato, sesamum, pulses, cotton, mesta, sugarcane, pineapple, citrus, apple, pear, peach, banana and medicinal plants. |
| 17 | Soils are shallow to very deep, loamy, red and lateritic, red and yellow with acidic in reaction. | Rice, millets, maize, potato, tea, coffee, rubber, orange, pine apple, jute, mustard, black gram, green gram and lentil. |
| 18 | Soils are clayey and slight to moderately sodic. | Rice, coconut, black gram, lentil, sunflower and groundnut. |
| 19 | Soils are deep, clayey red, lateritic, alluvium derived and strongly to moderately acidic in reaction. | Rice, tapioca, coconut and spices. |
| 20 | Soils are medium to very deep red-loamy, marine-derived, calcareous sandy with slightly to strongly acidic in reaction. | Rice, coconut, arecanut, oil palm, tapioca and pepper. |

Table S3. Descriptive statistics of available sulphur and micronutrients in soils the India (n = 242827)

| Nutrients | Minimum | Maximum | Mean | SD | CV (%) | Skewness | Kurtosis |
| --- | --- | --- | --- | --- | --- | --- | --- |
| Available S (mg kg^-1^) | 0.02 | 822 | 27.0 | 29.9 | 111 | 4.28 | 35.6 |
| Available Zn (mg kg^-1^) | 0.01 | 59.8 | 1.40 | 1.60 | 118 | 7.35 | 118 |
| Available B (mg kg^-1^) | 0.01 | 109 | 1.40 | 4.70 | 338 | 12.8 | 189 |
| Available Fe (mg kg^-1^) | 0.01 | 964 | 31.0 | 52.2 | 168 | 5.53 | 47.3 |
| Available Cu (mg kg^-1^) | 0.01 | 99.2 | 2.30 | 3.50 | 153 | 10.8 | 186 |
| Available Mn (mg kg^-1^) | 0.01 | 483 | 17.5 | 21.4 | 122 | 5.72 | 59.7 |

Note: SD = standard deviation and CV = coefficient of variation.


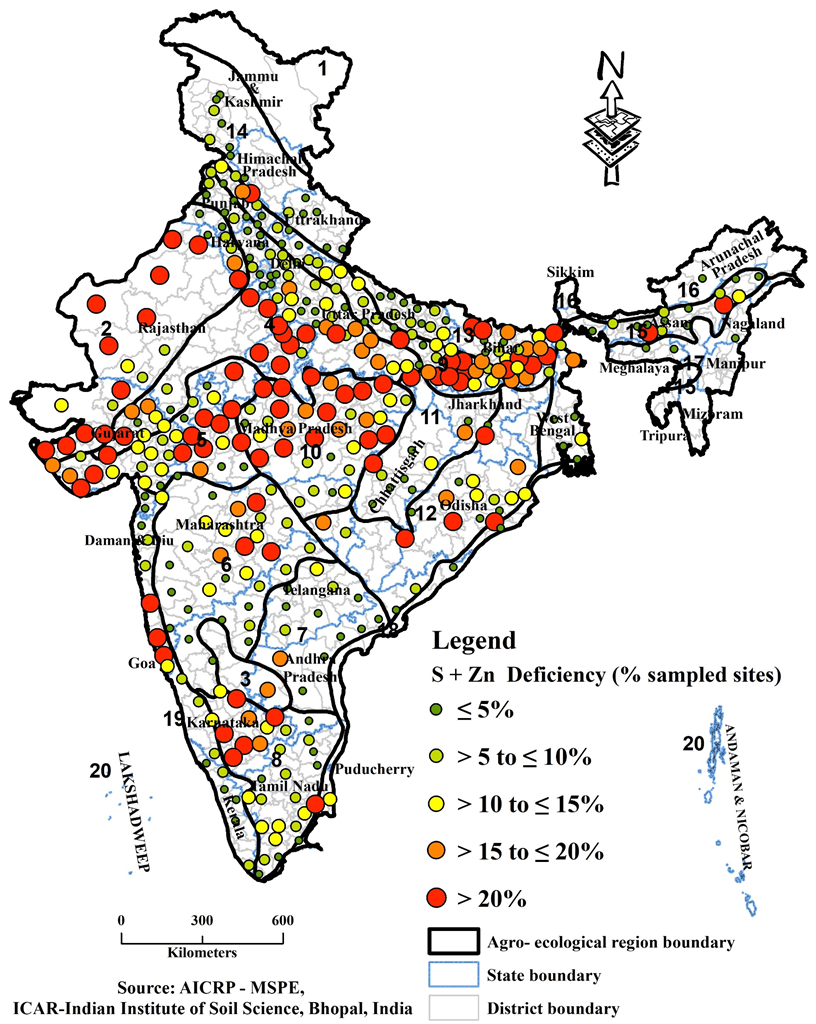


Figure S1. Distribution of S+ Zn deficiency in soils (% of soils) of different parts of India. Number denotes agro-ecological regions. (The figure has been prepared using ArcGIS software (version 10.5.1), Environmental Systems Research Institute, Redlands, California)


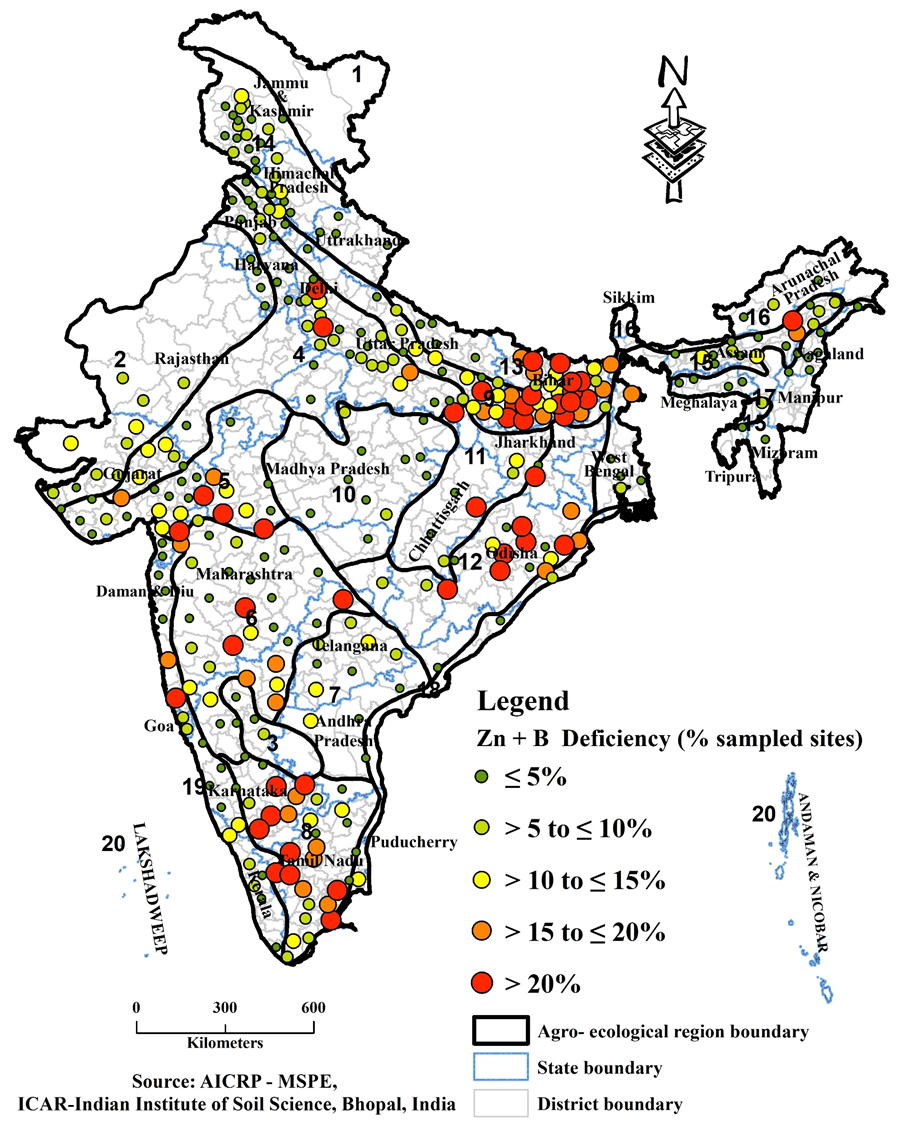


Figure S2. Distribution of Zn + B deficiency in soils (% of soils) of different parts of India. Number denotes agro-ecological regions. (The figure has been prepared using ArcGIS software (version 10.5.1), Environmental Systems Research Institute, Redlands, California)


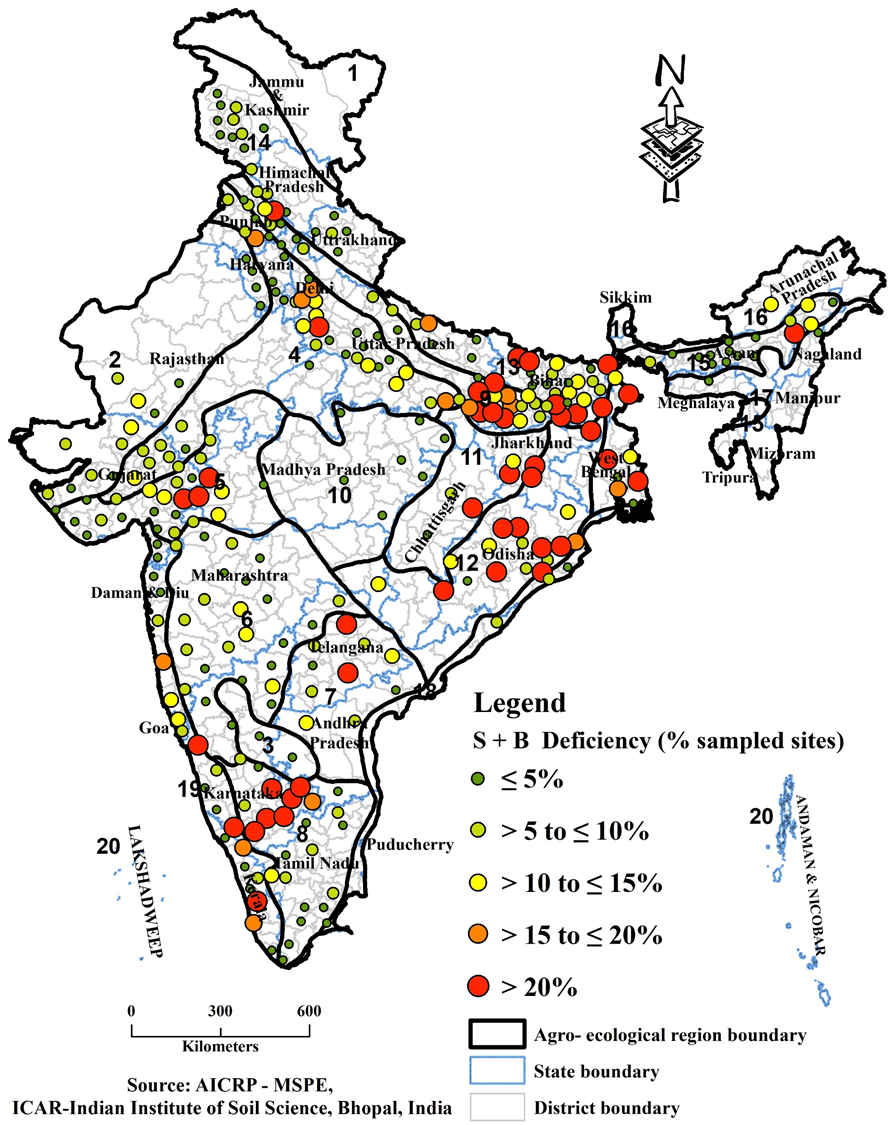


Figure S3. Distribution of S+ B deficiency in soils (% of soils) of different parts of India. Number denotes agro-ecological regions. (The figure has been prepared using ArcGIS software (version 10.5.1), Environmental Systems Research Institute, Redlands, California)


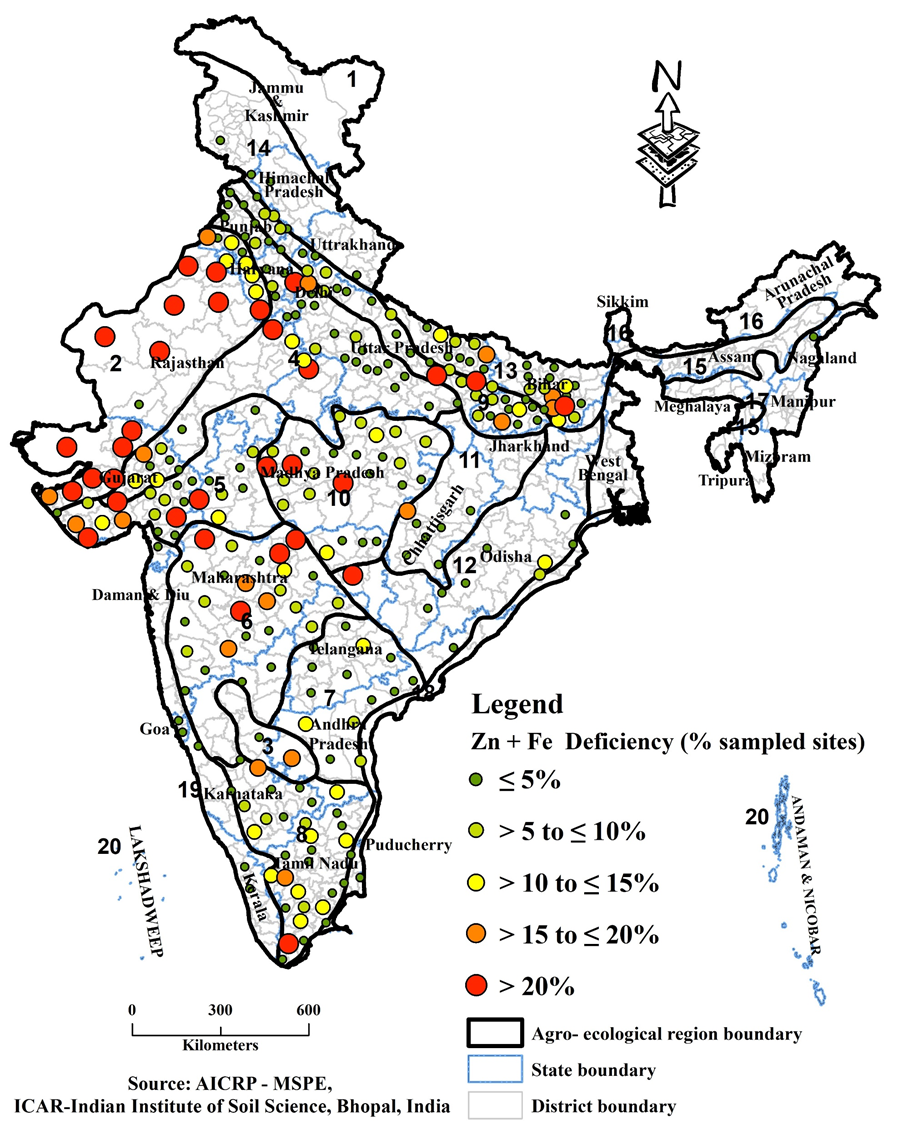


Figure S4. Distribution of Zn + Fe deficiency in soils (% of soils) of different parts of India. Number denotes agro-ecological regions. (The figure has been prepared using ArcGIS software (version 10.5.1), Environmental Systems Research Institute, Redlands, California)


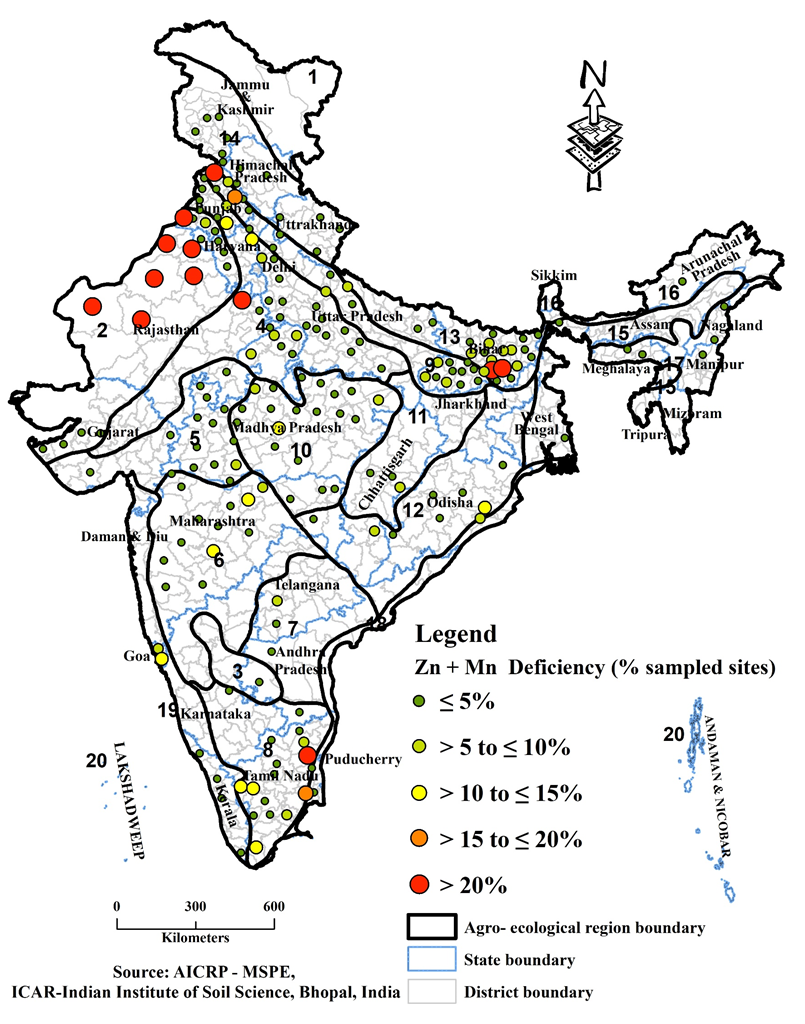


Figure S5. Distribution of Zn + Mn deficiency in soils (% of soils) of different parts of India. Number denotes agro-ecological regions. (The figure has been prepared using ArcGIS software (version 10.5.1), Environmental Systems Research Institute, Redlands, California)


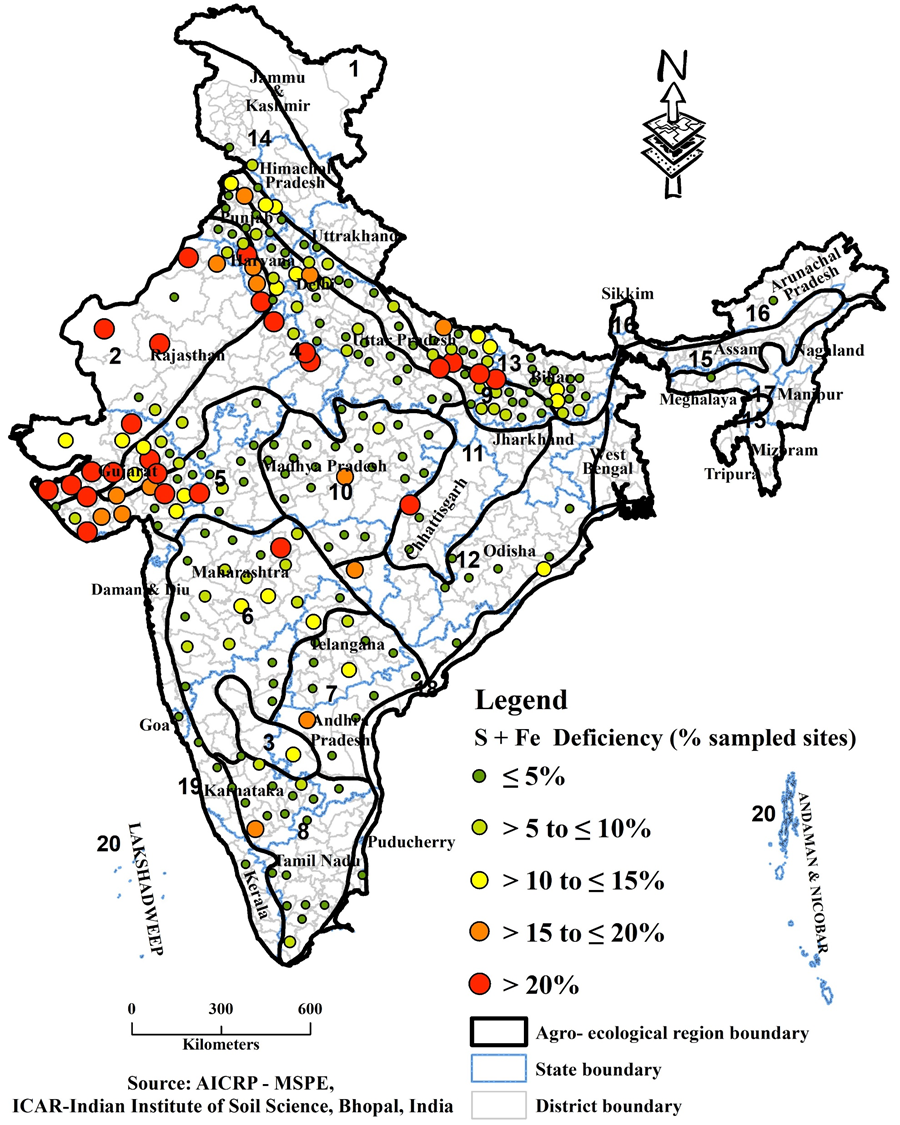


Figure S6. Distribution of S+ Fe deficiency in soils (% of soils) of different parts of India. Number denotes agro-ecological regions. (The figure has been prepared using ArcGIS software (version 10.5.1), Environmental Systems Research Institute, Redlands, California)


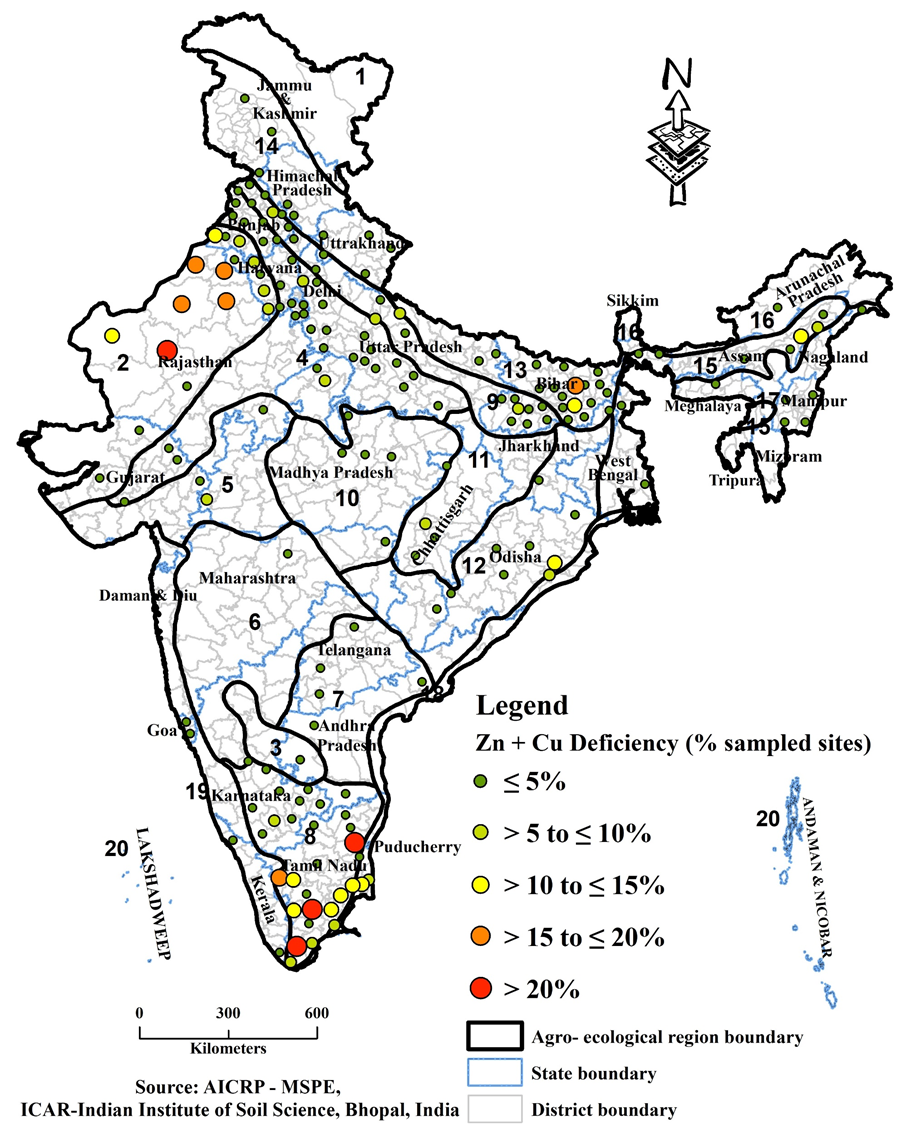


Figure S7. Distribution of Zn + Cu deficiency in soils (% of soils) of different parts of India. Number denotes agro-ecological regions. (The figure has been prepared using ArcGIS software (version 10.5.1), Environmental Systems Research Institute, Redlands, California)


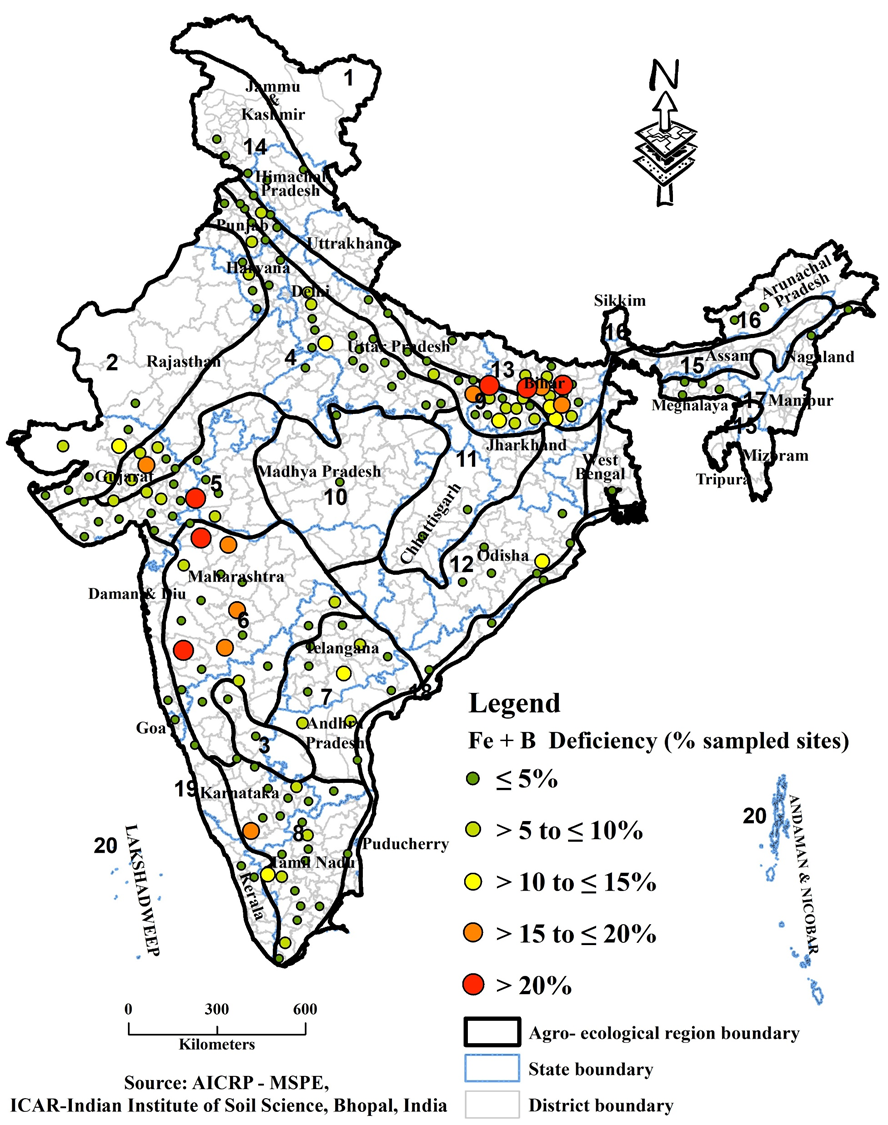


Figure S8. Distribution of Fe + B deficiency in soils (% of soils) of different parts of India. Number denotes agro-ecological regions. (The figure has been prepared using ArcGIS software (version 10.5.1), Environmental Systems Research Institute, Redlands, California)


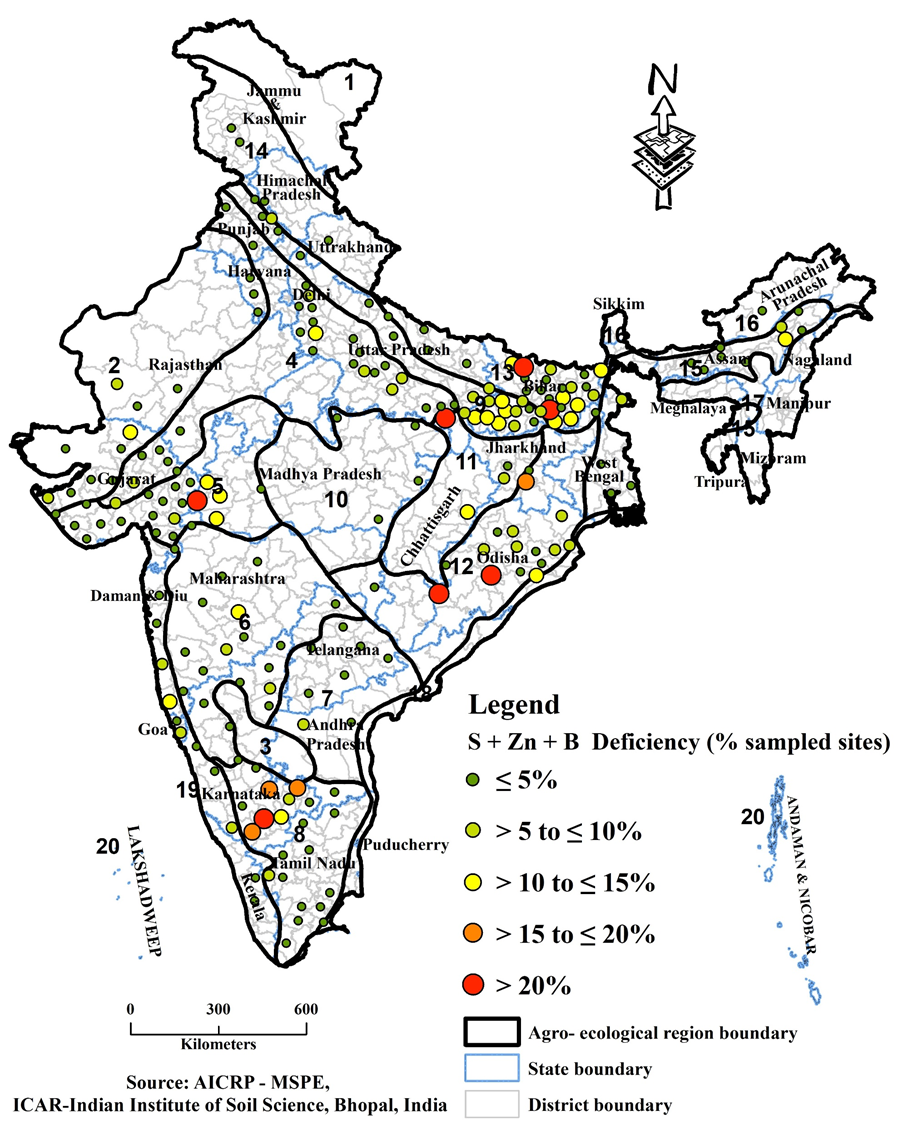


Figure S9. Distribution of S + Zn + B deficiency in soils (% of soils) of different parts of India. Number denotes agro-ecological regions. (The figure has been prepared using ArcGIS software (version 10.5.1), Environmental Systems Research Institute, Redlands, California)


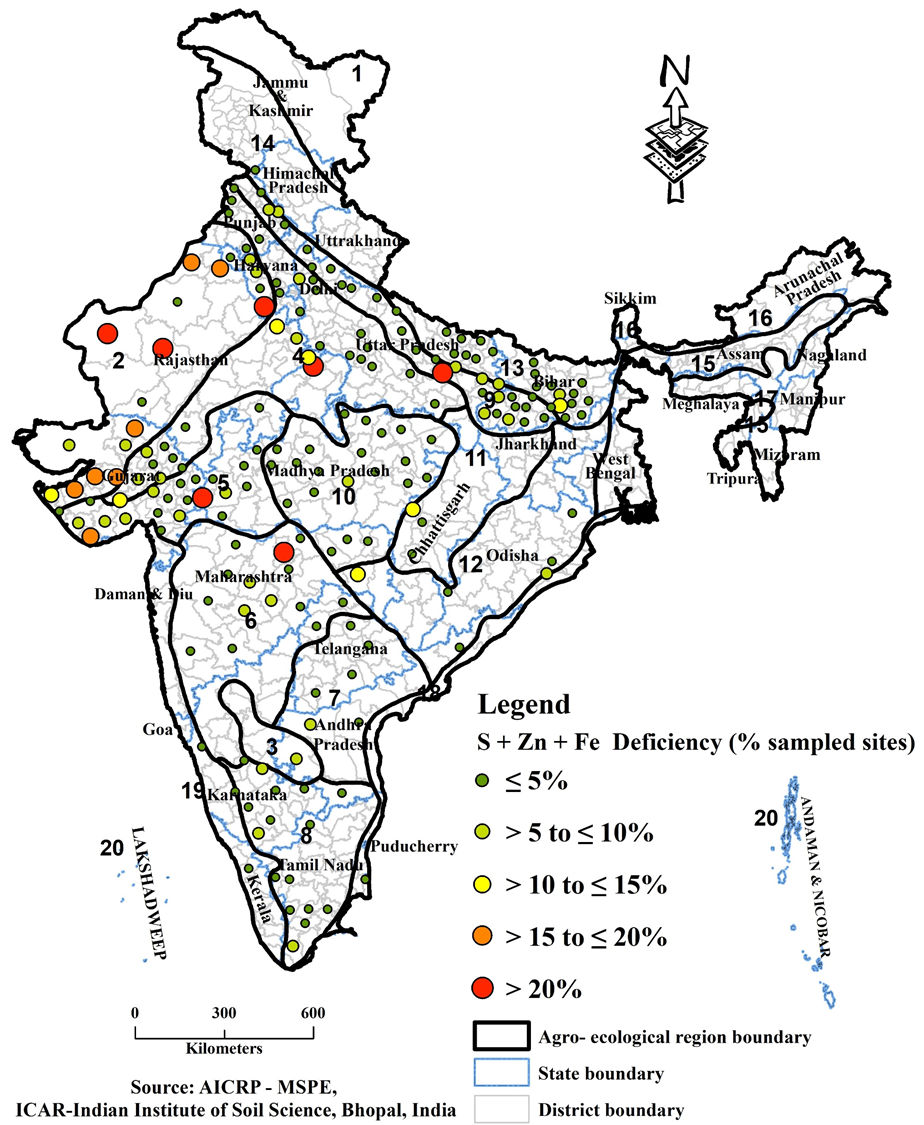


Figure S10. Distribution of S+ Zn + Fe deficiency in soils (% of soils) of different parts of India. Number denotes agro-ecological regions. (The figure has been prepared using ArcGIS software (version 10.5.1), Environmental Systems Research Institute, Redlands, California)


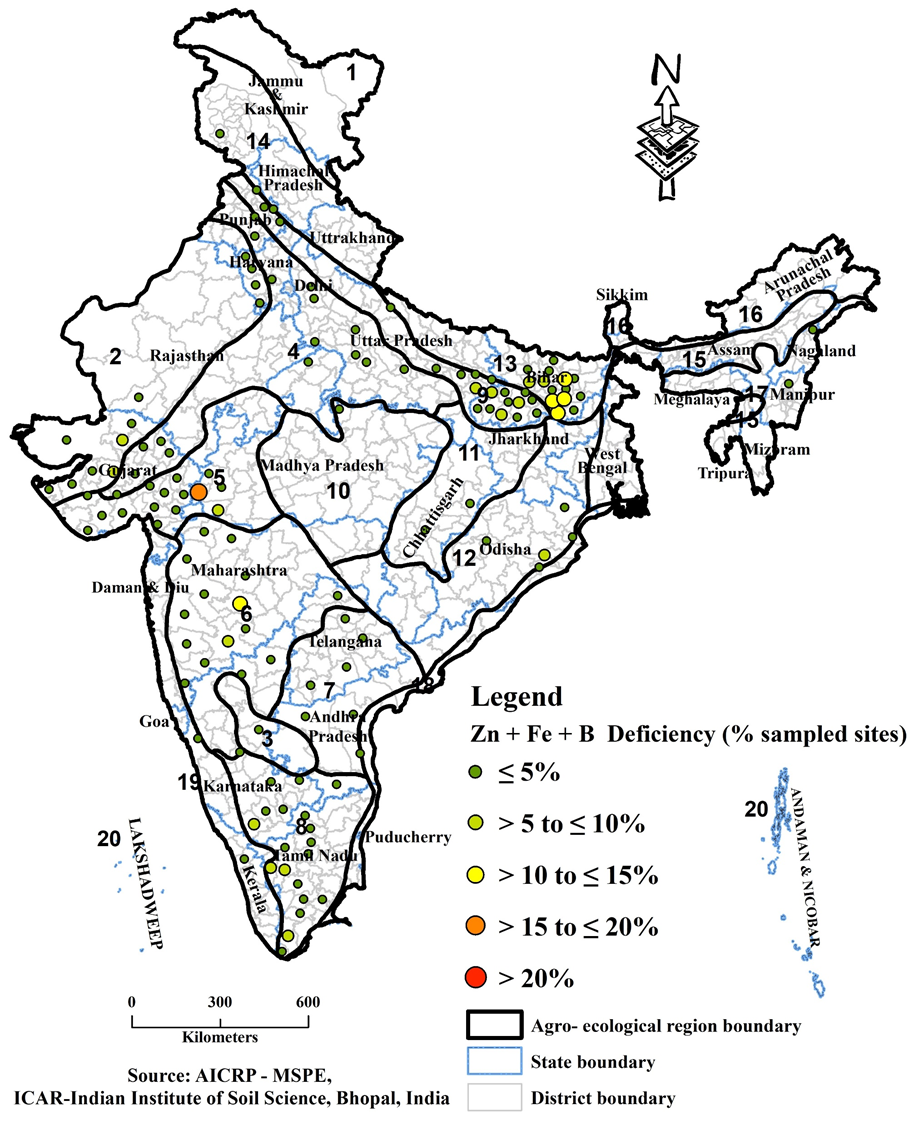


Figure S11. Distribution of Zn + Fe + B deficiency in soils (% of soils) of different parts of India. Number denotes agro-ecological regions. (The figure has been prepared using ArcGIS software (version 10.5.1), Environmental Systems Research Institute, Redlands, California)
